# Extranodal natural killer/T-cell lymphoma of the breast: a retrospective clinicopathological analysis of a consecutive 11-year case series

**DOI:** 10.1186/s13023-021-02110-x

**Published:** 2021-11-18

**Authors:** Wei Liu, Zihang Chen, Fanglan Li, Wenyan Zhang, Weiping Liu, Sha Zhao

**Affiliations:** 1 Department of Pathology, West China Hospital, Sichuan University, No. 37, GuoXue Xiang, Chengdu, 610041 Sichuan China; 2 Department of Pathology, Fujian Medical University Cancer Hospital, Fujian Cancer Hospital, Fuzhou, China; 3 Department of Nuclear Medicine, West China Hospital, Sichuan University, Chengdu, 610041 China

**Keywords:** Extranodal natural killer/T-cell lymphoma, Breast lymphoma, T-cell lymphoma, Epstein–Barr virus

## Abstract

**Background:**

Extranodal NK/T-cell lymphoma of the breast (ENKTL-Breast) is rarely detected in clinical practice, and its clinicopathological features remain unclear.

**Results:**

A consecutive 11-year (2010–2020) ENKTL-Breast case series was retrospectively analyzed. Eight cases of ENKTL-Breast were selected, with three primary and five secondary lesions, accounting for 3.5% (8/228) of all breast lymphomas. All patients were female with a median age of 46 years. Lesions presented as solid breast masses (8/8, 100%) and were usually located in the upper outer quadrant of the breast (7/8, 87.5%). B-symptoms were observed in seven (7/8, 87.5%) cases. Two primary ENKTL-Breast cases showed concomitant diseases (IgA nephropathy and chronic active hepatitis B). Histological and immunohistochemical features of ENKTL-Breast were similar to those of ENKTL at other sites. T-cell receptor rearrangement revealed clonality in all examined primary cases (2/2, 100%), but only in one secondary case (1/5, 20%). The disease progressed rapidly in two primary cases and both patients died within 3 and 9 months. For secondary cases, the disease manifested as a disseminated disease, with a median survival time of 6 months.

**Conclusions:**

Our data suggested that ENKTL-Breast clinically mimics breast cancer to some extent, though B-symptoms might serve as a distinguishing factor. ENKTL-Breast is highly aggressive and patients with this disease exhibit a short survival time. Primary ENKTL-Breast tends to originate from activated cytotoxic T-cells, and immune-related diseases may be involved in its pathogenesis and development.

**Supplementary Information:**

The online version contains supplementary material available at 10.1186/s13023-021-02110-x.

## Background

Extranodal natural killer/T-cell lymphoma (ENKTL) is an uncommon malignancy, which is prevalent in East Asia and Central and South America and is highly associated with Epstein–Barr virus (EBV) [1–5]. In ENKTL, the upper aerodigestive tract is most involved, with the nasal cavity being the prototypical site of involvement. The disease may also involve only extranasal sites, such as the skin, subcutaneous tissues, gastrointestinal tract, and testes. However, it rarely involves the breast [1]. In fact, only a few cases of ENKTL of the breast (ENKTL-Breast) have been reported so far [6–12]. Even in East Asia, where a higher incidence of ENKTL is observed, there are only sporadic cases of ENKTL-Breast. As a result, systematic analysis and data of ENKTL-Breast are lacking [5].

ENKTL involves different extranasal sites and presents distinct clinicopathological features depending on the primary site of involvement. For instance, gastrointestinal lesions often present with perforation or bleeding, laryngeal lesions can mimic inflammation or well-differentiated squamous cell carcinoma [13], and neoplastic cells of testicular lesions may show aberrant CD20 expression [14]. Thus, we hypothesized that ENKTL-Breast may have distinct clinicopathologic features that differentiate it from other involved sites; consequently, we retrospectively analyzed the clinicopathological features of eight patients with ENKTL-Breast in a single-center study. We believe that this study will aid clinicians and pathologists to better recognize this rare ENKTL presentation.

## Results

### Clinical features

Eight cases of ENKTL-Breast, including three primary and five secondary cases, were selected for this study. The clinical features of these cases are summarized in Table 1. In the secondary ENKTL-Breast cases, two patients (cases 5 and 6) had a history of nasal ENKTL, one patient (case 4) had primary vaginal ENKTL, one patient (case 8) showed simultaneous involvement of the breast and gastrointestinal tract, and the remaining patient (case 7) did not have sufficient data to confirm primary ENKTL-Breast.

**Table 1:** Clinical features of ENKTL-Breast cases

Case	P/S	Age/sex	Side	Site	Size (cm)	Other sites involvement	BM	Stage	B-symptoms	LDH (IU/L)	EBV –DNA (copies/ml)	Concomitant diseases	Treatment	Follow-Up*
1	P	31/F	R	areola	5.7 × 3.5	An isolated right axillary node involvement at the diagnosis of primary ENKTL-Breast	Normal	I	Yes	311	1.81 × 10^4^	IgA nephropathy	GemxoD + Pe	Died (9 mo)
2	P	26/F	R	UOQ	4.0 × 3.0	Both side of the lungs and the liver were involved one and a half months after the diagnosis of primary ENKTL-Breast	Normal	I	Yes	787	1.40 × 10^3^	Chronic active hepatitis B	GDP	Died (3 mo)
3	P	54/F	L	UOQ	2.0 × 2.0	None	Normal	I	No	NA	NA	NA	NA	LTF
4	S	30/F	L	UOQ	2.5 × 2.0	Breast involvement appeared 20 months after the diagnosis of primary vagina ENKTL	Normal	IV	Yes	449	1.30 × 10^4^	None	GLIDE + RT, followed by GMOX + Ca	Died (6 mo)
5	S	57/F	R	UOQ	1.0 × 1.0	Breast involvement appeared 8 months after the diagnosis of primary nasal ENKTL	Normal	IV	Yes	160	Negative	None	VDLP, followed by SMILE + RT	Died (5 mo)
6	S	46/F	R	UOQ	2.8 × 2.0	Breast involvement appeared 12 months after the diagnosis of primary nasal ENKTL	NA	IV	Yes	NA	NA	None	NA	LTF
7	S	63/F	B	UOQ	R,2.0 × 2.0 L,5.0 × 3.0	NA	NA	ND	Yes	NA	NA	None	NA	Died (11 mo)
8	S	39/F	R	UOQ	2.0 × 1.0	Simultaneous involvement of GI tract and the breast	NA	IV	Yes	982	NA	Graves’ disease	NA	Died (2 mo)

^*^The Follow-Up time was calculated from the time of diagnosing ENKTL-Breast. The stage was measured at the time of diagnosing ENKTL-breast

Patients with a history of ENKTL had just an isolated lesion (stage I) at the time of diagnosis from the primary site

B, bilateral; BM, bone marrow; Ca, Camrelizumab; F, female; GDP, gemcitabine + cisplatin + dexamethasone; GemxoD, gemcitabine + oxaliplatin + dexamethasone; GLIDE, gemcitabine + L-asparaginase + ifosfamide + dexamethasone; GMOX, gemcitabine + oxaliplatin; GI, gastrointestinal; L, left; LTF, lost to follow-up; mo, month; NA, not available; ND, not done; Pe, pegaspargase; P, Primary; R,right; RT, radiotherapy; S, Secondary; SMILE, methotrexate + dexamethasone + fosfamide + pegaspargase + etoposide + mesna; UOQ, upper outer quadran; VDLP, etoposide + cisplatin + dexamethasone + pegaspargase

**Fig. 1:**
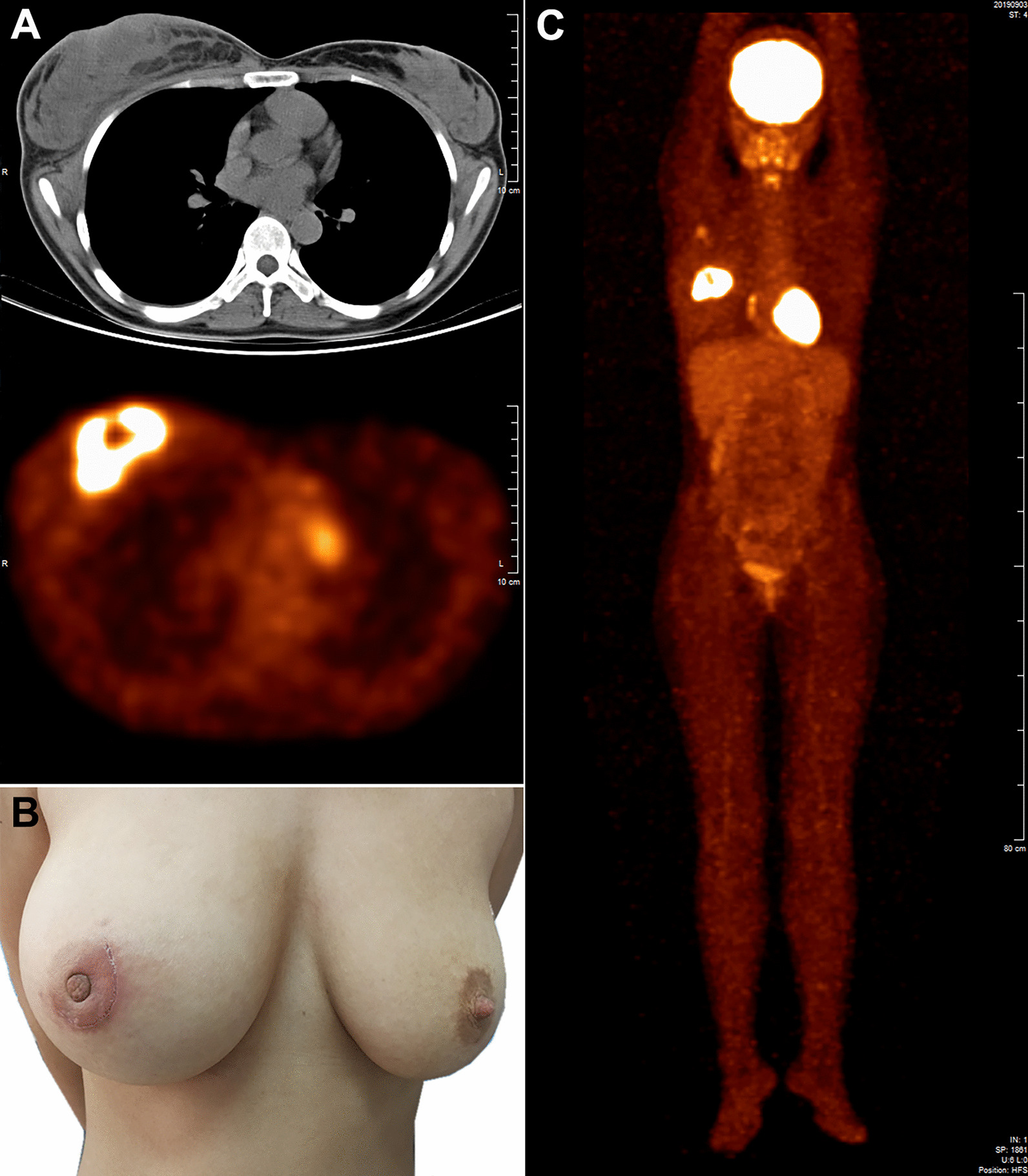
Clinical and radiological features of ENKTL-Breast (Case 1). **a** A CT scan showed that the lesion presented as a soft tissue density mass with a diameter of 6.6 cm in the right breast with unclear boundaries. **b** The right breast was significantly swollen and enlarged, and the nipple was indented. The mass located below the areola was partially excised for diagnosis (surgical incisions are apparent around the areola). **c** PET-CT showed increased F-FDG uptake by the breast lesion and ipsilateral axillary lymph node (stage IE)

All patients were female, with a median age of 46 years (range 26–63 years), and most presented with B-symptoms (7/8, 87.5%). From the radiological results, all lesions presented as solid masses (Fig. 1a) with a median length of 2.5 cm (range 1.0–5.7 cm) in the greatest dimension, with five cases occurring on the right side (5/8, 62.5%), and seven cases located in the upper outer area of the breast (7/8, 87.5%). Most cases (7/8, 87.5%) presented with a unilateral lesion while one secondary case (case 7) showed bilateral involvement. Only one case (case 3) had cutaneous infiltration leading to formation of an ulcer. All patients were clinically suspected of having breast cancer at the initial hospital visit. In addition, one of the patients showed enlargement and swelling of the breast (case 1; Fig. 1b). In the laboratory tests, elevated lactate dehydrogenase levels and plasma EBV-DNA loads were detected in 80% (4/5) and 75% (3/4) of the patients who undertook the test, respectively. Seven cases were available for staging, of which three were stage I (Fig. 1c) and the other four were stage IV.

Additionally, two primary cases and one secondary case showed concomitant diseases. Case 1 (primary case) had a history of IgA nephropathy (stage IV) for 8 years, case 2 (primary case) had a history of chronic active hepatitis B with abnormal liver function and elevated hepatitis B virus (HBV)-DNA load (2.4 × 10^6^ IU/mL), and case 8 (secondary case) had Graves’ disease for 10 years.

### Morphological features

**Fig. 2:**
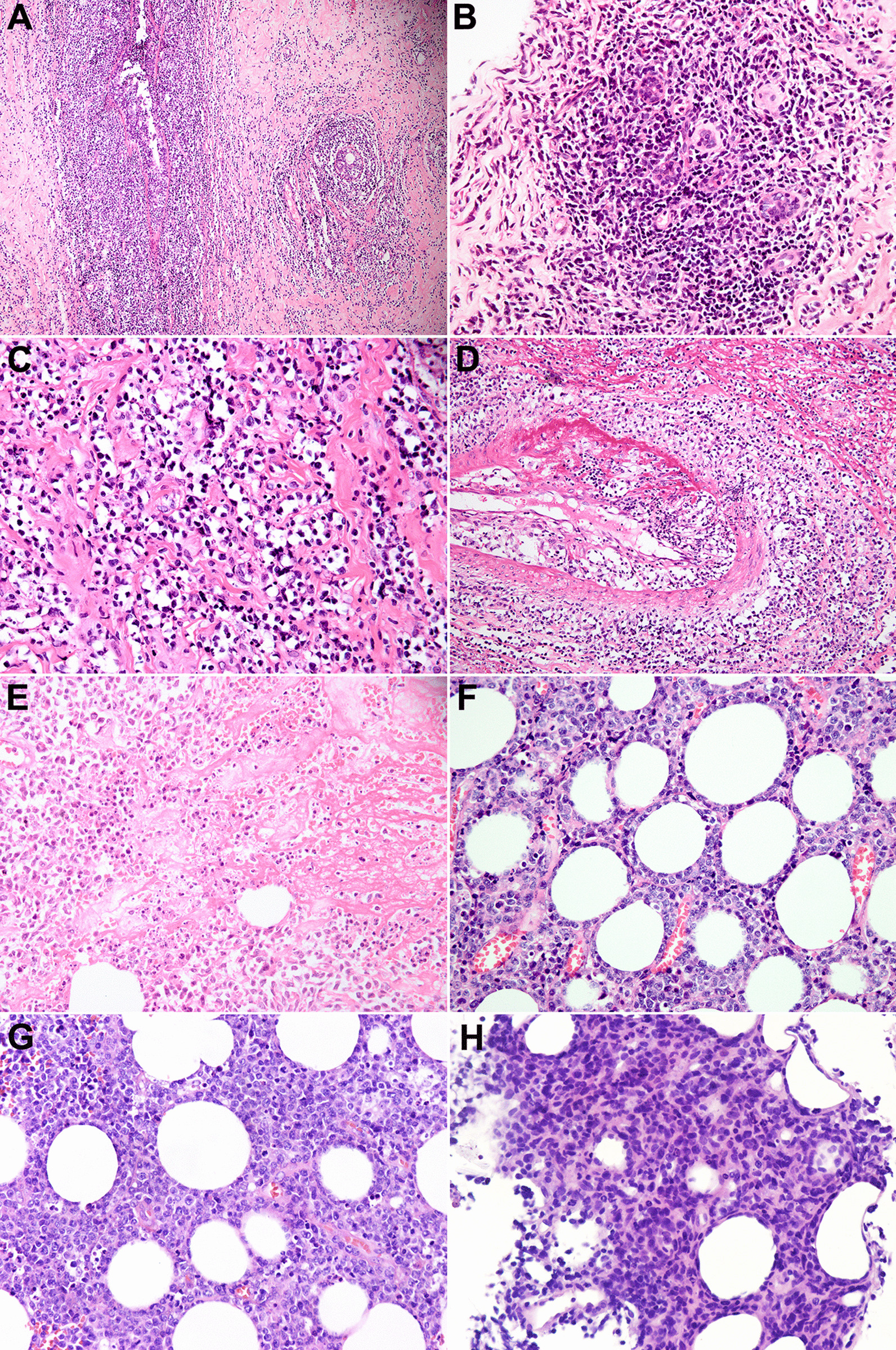
Morphological features of ENKTL-Breast (hematoxylin and eosin statin). **a** Diffuse dense infiltration of lymphoid cells in mammary ducts and surrounding stroma, forming lymphoepithelial lesions (× 100 magnification). **b** Lymphoma cells infiltrated the lobules (× 200 magnification). **c** Tumor cells infiltrated the specialized breast stroma (× 400 magnification). **d** Angiocentric and angiodestructive growth patterns (× 200 magnification). **e** Patches of coagulative necrosis and apoptotic bodies are apparent (× 400 magnification). **f** Neoplastic cells involved subcutaneous fat lobules of the breast, partly with sparing of septa and rimed fat spaces (× 400 magnification). **g** Monomorphic large cells (× 400 magnification). **h** Pleomorphic tumor cells (× 400 magnification)

Morphologically, the primary and secondary ENKTL-Breast cases were similar. The histopathological features are summarized in Table 2. All eight cases showed lymphoid cell infiltration in the mammary ducts (Fig. 2a), lobules (Fig. 2b), and stroma (Fig. 2c), and formation of lymphoepithelial lesions (Fig. 2a). An angiocentric and/or angiodestructive tumor growth pattern was also detected (Fig. 2d). Patchy or cluster necrosis (6/8, 75%) and apoptotic bodies (8/8,100%) were observed (Fig. 2e). Neoplastic cells involved subcutaneous fat lobules, partly with septa sparing and rimed fat spaces (8/8, 100%; Fig. 2f). Most cases (6/8, 87.5%) displayed apparent heterogeneity of pleomorphic tumor cells (Fig. 2h) in medium-to-large or large size, with irregular or twisted nuclei, granular chromatin, and inconspicuous or small nucleoli. Further, prominent nucleoli and vesicular nuclei were also found in some large cells. Two monomorphic cases (cases 5 and 7) were composed of small-to-medium and large cells, respectively (Fig. 2g). Mitoses were commonly observed in all cases.

**Table 2:** Morphological features of ENKTL-Breast cases

Case	Biopsy	Skin ulcer	PEH	Infiltration area	Angiocentric/angiodestructive	Patchy necrosis	Apoptotic bodies	Cell size	Shape of cell
1	Excision	−	−	Breast parenchyma	+	+	+	Medium-large	Pleomorphic
2	Core needle biopsy	−	−	Breast parenchyma	+	+	+	Large	Pleomorphic
3	Excision	+	−	Breast parenchyma and overlying epithelium	+	+	+	Large	Pleomorphic
4	Core needle biopsy	−	−	Breast parenchyma	+	+	+	Medium	Pleomorphic
5	Core needle biopsy	−	−	Breast parenchyma	+	−	+	Small-medium	Monomorphic
6	Core needle biopsy	−	−	Breast parenchyma	+	−	+	Medium	Pleomorphic
7	Excision	−	−	Breast parenchyma	+	+	+	Large	Monomorphic
8	Excision	−	−	Breast parenchyma	+	+	+	Medium-large	Pleomorphic

PEH, pseudoepitheliomatous hyperplasia; + , positive; − , negative

### Immunohistochemical phenotype and molecular findings

**Fig. 3:**
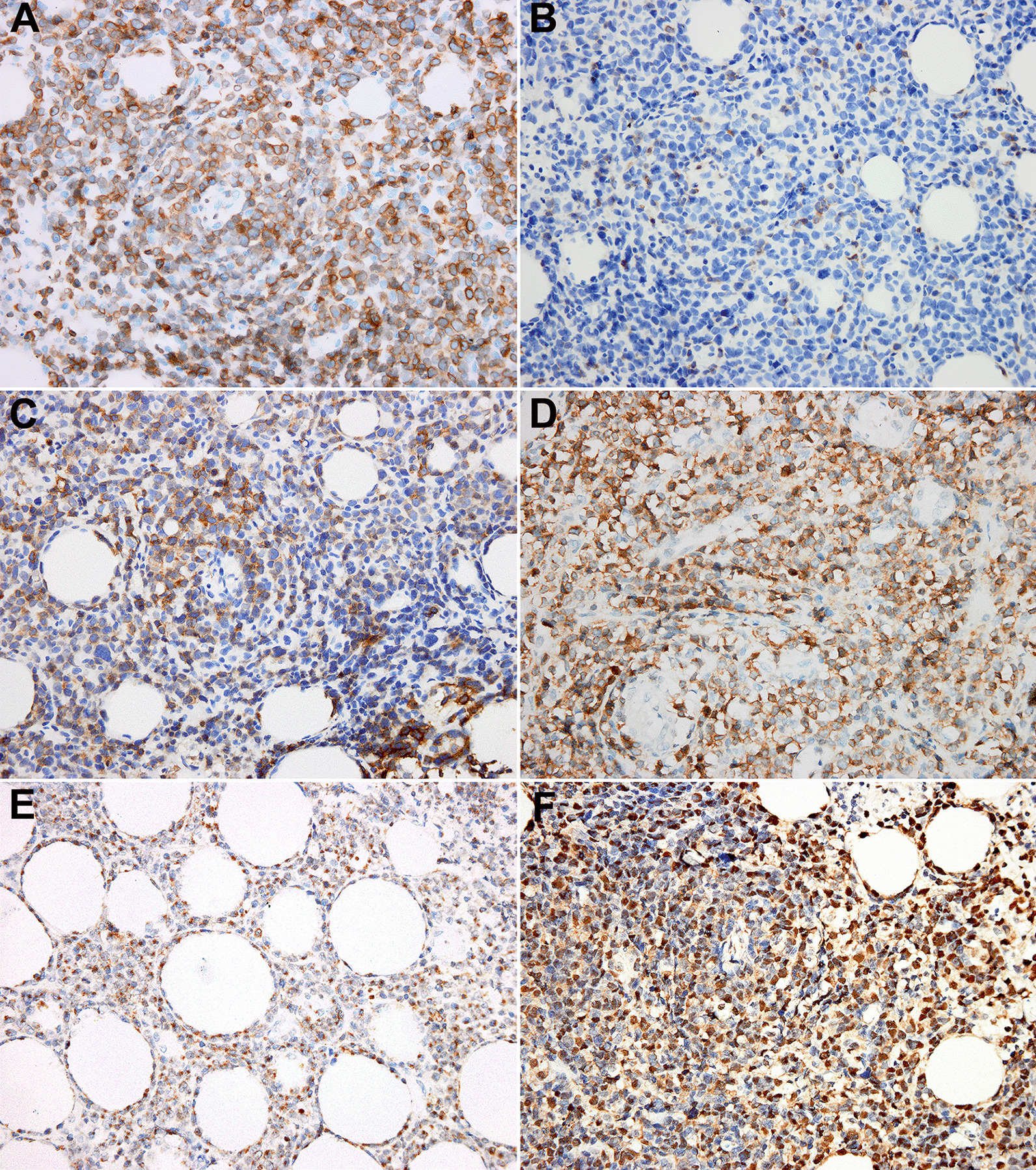
Immunophenotype and in situ hybridization features of ENKTL-breast. **a** CD3; **b** CD5; **c** CD30; **d** CD56; **e** TIA-1; **f** EBER (× 400 magnification)

All cases were positive for CD3 (Fig. 3a) and cytotoxic granules (TIA-1/GrB; Fig. 3e), and negative for CD20, CD5 (Fig. 3b), CD4, and CD8. CD56 (Fig. 3d) was positive in most cases (6/8, 75%). Six cases were positive for CD30 (median: 62.5%; range 20–80%; Fig. 3c). Median proliferation index Ki-67 was 80% (range 40–90%). Seven cases were tested for T-cell receptor (*TR*)-γ rearrangement, of which two primary cases and one secondary case were monoclonal. EBV-encoded small RNAs (EBER) was positive for all cases (range 40–80%; Fig. 3f). The abovementioned data are summarized in Table 3.

**Table 3:** Immunohistochemical analysis, EBER, and TR-γ rearrangement study of ENKTL-Breast cases

Case	cCD3	CD20	CD2	CD5	CD7	CD4	CD8	TIA-1	GrB	CD56	CD30	Ki-67	EBER	TR-γ
1	+	−	ND	−	ND	−	−	+	+	−	55%	75%	+	+
2	+	−	+	−	−	−	−	+	+	+ , partly positive	70%	80%	+	+
3	+	−	ND	−	ND	ND	ND	+	+	+	80%	85%	+	ND
4	+	−	ND	−	ND	−	−	+	+	−	20%	85%	+	−
5	+	−	ND	−	ND	−	−	+	+	+	0	80%	+	+
6	+	−	ND	−	ND	ND	ND	+	+	+	0	40%	+	−
7	+	−	+	−	+	−	−	+	+	+	75%	80%	+	−
8	+	−	+	−	+	−	−	+	+	+	20%	90%	+	−

cCD3, cytoplasmic CD3; GrB, granzyme B; ND, not done; TR-γ, T-cell receptor-γ gene rearrangement; EBER, EBV-encoded small RNAs; + : positive/clonal TR-γ rearrangement; − : negative/not clonal TR-γ rearrangement

### Follow-up and survival analysis

Six cases were available for follow-up and survival analysis (two primary and four secondary). For the two primary cases, although the patients were at stage I at the time of diagnosis (radiologically confirmed), the disease progressed rapidly and involved multiple organs in a short span of time. These patients responded poorly to chemotherapy and died of the disease within 9 and 3 months, respectively. For the four secondary cases, many organs other than the breast were also involved and showed wide dissemination. These patients also had a poor response to chemotherapy and/or radiotherapy, with a median OS of only 6 months (range 2–11 months).

### Review of the reported cases of ENKTL-breast

We identified seven cases of ENKTL-Breast (both primary and secondary) with sufficient clinicopathological data acquired from literature review [6–11]. The clinicopathological features of these cases and those of our study are summarized in Table 4 (also see Additional file 1: Table S1, which summarizes these features in detail). A total of 15 cases (including our cohort and cases from the literature) were collected, including seven primary cases and eight secondary cases. Nine out of 12 (75%) of the patients were Asian. All patients were female and had a median age of 41 years (range 20–63 years). Majority of the cases showed B-symptoms (10/12, 83.3%) and initially presented with a breast mass (14/15, 93.3%). Beside the three cases from our cohort that presented with a coexistent disease, three cases in the literature also reported heart transplantation, saline breast implants, and hypothyroidism, respectively. Compared with the secondary cases (1/6, 16.7%), primary cases tended to have clonal *TR* rearrangement (3/5, 60%). Of the 10 patients with survival data, 90% (9/10) died of the disease, and had a median survival of 5 months.

**Table 4:** Clinical features of ENKTL-Breast cases acquired from the present study and literature review

Characteristics	Present study	Literature [6–11]	Total
Number of cases	8 (P: 3; S: 5)	7 (P: 4; S: 3)	15 (P: 7; S:8)
Median age (range) (year)	46 (26–63)	38 (20–47)	41 (20–63)
Origin (Asian/non-Asian)	8/0	1/3	9/3
Initial presented with breast mass (yes/no)	8/0	6/1	14/1
B symptoms (yes/no)	7/1	3/1	10/2
Ann Arbor stage (I/II vs III/IV)	P: 3/0; S: 0/4	P: 2/0; S: 0/3	P: 5/0; S: 0/7
Immunophenotype CD56 (positive/negative)	P: 2/1; S: 4/1	P: 2/0; S: 2/1	P: 4/1; S: 6/2
EBER (positive/negative)	8/0	6/1	14/1
TR gene rearrangement (monoclonal/polyclonal)	P: 2/0; S: 1/4	P: 1/2; S: 0/1	P: 3/2; S: 1/5
Concomitant diseases (yes/no)	P: 2/0; S: 1/4	P: 3/0; S: 0/3	P: 5/0; S: 1/7
Outcome(died/alive)	6/0	3/1	9/1
Median survival (range) (month)	6 (2–11)	5 (1–18)	5 (1–18)

NA, not available; P, primary cases; S, secondary cases, mo, months; EBER, EBV-encoded small RNAs; TR, T-cell receptor

## Discussion

Non-Hodgkin lymphoma (NHL) of the breast is uncommon, accounting for approximately 5% of all breast malignancies [15–18]. According to the literature review, the most common histological breast lymphoma type is diffuse large B-cell lymphoma. T-cell or NK/T-cell lymphomas that involve the breast are uncommon. Both primary and secondary ENKTL usually present with a breast mass and clinical findings concerning for breast cancer. Herein, we summarize clinicopathological features of ENKTL presenting in the breast. To the best of our knowledge, this is the largest cohort of ENKTL-Breast reported to date.

The clinicopathological features of ENKTL-Breast cases showed many similarities between our cases and previously reported cases. We also presented the characteristics of primary ENKTL-Breast cases and ENKTL at other primary sites in Table 5. Clinically, primary ENKTL-Breast mainly occurs in young to middle-aged (median: 40 years, range 20–54 years) female patients. The age of onset of primary ENKTL-Breast was similar to those of primary extra-facial-midline ENKTL (gastrointestinal tract, skin, and testis). However, primary facial midline ENKTL (nasal and larynx) often occurred in patients older than 50 years old. The patients typically presented with a mass in the upper outer quadrant of the breast (other than ulceration); however, they were distinct from primary ENKTL at other sites, which led to the initial consideration of breast cancer. Nevertheless, primary ENKTL-Breast patients were more likely to suffer from B-symptoms, and this could be a possible clue for physicians to think of a differential diagnosis. Notably, in East Asia and Central/South America, where ENKTL is more common, physicians should be aware of this condition, since ENKTL is also one of the common types of breast lymphomas (the 4^th^ most common type in our institution). Interestingly, both in our cohort and in cases queried from the literature, primary cases showed immune-related concomitant diseases or conditions, including immunosuppression status (heart transplantation) [7], autoimmune diseases (IgA nephropathy, hypothyroidism) [9], and other diseases or conditions that may have affected immune function (chronic active HBV infection, saline breast implant) [8]. Similarly, several breast lymphoma case series have reported a high prevalence of antecedent autoimmune diseases, such as Hashimoto’s thyroiditis (19–30%) [19, 20]. In addition, an increased risk of NHL in connective tissues and autoimmune diseases has also been reported [21, 22]. It is suggested that immune-related diseases may play a potential role in primary ENKTL-Breast, although robust data of such patients are lacking. We speculate that this may be because patients with immune-related diseases or conditions are more prone to EBV infection/reactivation, and this long-lasting activation may further promote the occurrence of ENKTL. Thus, more cases and fundamental research are needed to clarify the relationship between immune-related diseases or conditions and primary ENKTL-Breast.

**Table 5:** Clinicopathological features of ENKTL of different primary sites

Characteristics	Breast*, n (%)	Nasal, n (%) [23]	GI tract, n (%) [24]	Skin, n (%) [25]	Testis, n (%) [14]	Larynx, n (%) [13]
Number of cases	7	92	55	16	21	31
Median age (range) (year)	40 (20–54)	52 (21–89)	39 (14–75)	32 (16–72)	44 (21–79)	50 (13–77)
B symptoms	5 (100)	36 (39)	19 (35)	11 (69)	7 (33)	10 (32)
Ulceration	0 (0)	NR	55 (100)	7 (44)	NR	12 (39)
Concomitant diseases	5 (100)	NR	NR	NR	NR	NR
Pleomorphic	5 (83)	NR	42 (76)	13 (81)	NR	8 (26)
Number of CD30 + cases	4 (67)	36 (39)	13 (41)	2 (15)	5 (31)	NR
Median CD30 expression, % (range)	62.5 (50–80)	NR	NR	NR	NR	NR
Median Ki-67 index, % (range)	80 (70–95)	NR	70 (50–90)	60 (NR)	80 (50–90)	60 (30–80)
TR gene rearrangement (monoclonal/polyclonal)	3 (60)	35 (38)	9 (53)	0 (0)	NR	1 (17)
Median survival (range) (month)	5 (1–9)	19.2 (NR)	14 (1–56)	7 (2–20)	15.3 (0.5–87)	9 (1–104)

^*^Including primary cases from the current study and the literature review in Additional file 1: Table S1

NR, not report; TR, T-cell receptor; n, number

Morphologically, pleomorphic tumor cells diffusely infiltrate the breast parenchyma, similar to nasal and extranasal ENKTL. In all our cases, the neoplastic cells were positive for CD3 and TIA1, with a high Ki-67 index (median: 80%, range 70–95%) and negative for CD5, in accordance with other extranasal ENKTL. Primary ENKTL-Breast tended to express CD30 (4/6, 67%) at a relatively high positive rate (median: 62.5%, range 50–80%). Notably, primary lesions were likely to have monoclonal *TR* rearrangements (3/5, 60%). In our cohort, neoplastic cells of two primary ENKTL-Breast cases (2/2, 100%) that underwent *TR* rearrangement showed monoclonality with a cytotoxic T-cell phenotype. In addition, 33% of the primary ENKTL-Breast cases from the literature review also reported a monoclonal *TR* gene rearrangement [8]. Clonal *TR* gene rearrangement has been reported in 10–40% of ENKTL, presumably because of cytotoxic T lymphocyte origin [1]. Additionally, a large case series from the MD Anderson Cancer Center has also reported that extranasal ENKTL cases were more likely to carry monoclonal *TR* gene rearrangements (extranasal type: 80%, 4/5 vs. nasal type: 27%, 4/15) [12]. Therefore, examining normal T-cell populations and further studying these malignancies in the breast may help in elucidating the origin and behavior of these exceedingly rare lymphomas.

In our series, primary ENKTL-Breast had a poor prognosis with a median OS of only 5 months (range 1–9 months), which is much shorter than that reported for the nasal ENKTL cohort by the International Peripheral T-cell Lymphoma Project (19.2 months) [23]. Moreover, its median OS was also the lowest among the extranasal ENKTL cohorts with other common sites (GI tract, skin, and testis) analyzed by both our institution and other hospitals (median OS: 7–15.3 months) [14, 24, 25]. This may have been due to the aggressive behavior of the disease, i.e., rapid dissemination to other sites and chemotherapy resistance even in patients with early stage disease. It may be argued that the disease is probably already disseminated from the beginning; therefore, a comprehensive assessment and close monitoring is required for such primary ENKLT-Breast cases. Similarly, we observed that the prognosis was poor for the secondary ENKTL-Breast cases, with a median OS of 6 months (range 2–18 months). Both our results and data from the literature review demonstrated that breast involvement implied advanced stage ENKTL with multiorgan involvement. Therefore, whole-body images for measuring disease involvement followed by effective treatment strategies are urgently needed. It is encouraging that novel therapies, such as immunotherapy (PD1/PDL1 inhibitor) and other targeted therapies (anti-CD30 therapy), have been shown to prolong the OS in relapse and refractory ENKTL cases [26–28]. Perhaps, these therapies can bring hope to patients experiencing ENKTL-Breast.

## Conclusions

In conclusion, both primary and secondary ENKTL-Breast cases are rare neoplastic diseases that often present with solid masses in the breast parenchyma (other than ulceration), whereby its growth pattern and morphology are similar to those of primary ENKTL at other sites. Primary ENKTL-Breast was more likely to have a monoclonal *TR* rearrangement. Immune-related diseases and EBV reactivation may play a potential role in the pathogenesis. Moreover, both primary and secondary ENKTL-Breast cases present a highly aggressive clinical course, short survival time, and poor response to therapy. Thus, more study is needed to unravel the underlying etiology and provide new therapeutic options for patients with ENKTL-Breast.

## Materials and methods

### Case selection

In total, 228 patients were diagnosed with breast lymphoma (including primary and secondary lesions) between 2010 and 2020 based on data acquired from the database of the Department of Pathology, West China Hospital, Sichuan University. Histologically, the most common lymphoma type was diffuse large B-cell lymphoma, not otherwise specified (176/228, 77.2%), followed by extranodal marginal zone lymphoma of mucosa-associated lymphoid tissue (MALT lymphoma; 11/228, 4.8%), and lastly B-lymphoblastic leukemia/lymphoma (11/228, 4.8%) (Additional file 1: Table S2). Eight cases (8/228, 3.5%) of ENKTL-Breast were identified, which was the 4th most common type of lymphoma occurring in the breast. All tissue sections were reviewed independently and re-diagnosed by three hematopathologists (W.Z., W.L., and S.Z.) according to the World Health Organization classification of tumors of hematopoietic and lymphoid tissues (Revised 4th Edition, 2017) [1]. Detailed clinical data, such as age, sex, clinical course, symptoms, laboratory tests, imaging findings, and treatment details, were collected from electronic medical records. Follow-up data were obtained by telephone interviews and/or medical records. Overall survival (OS) was calculated from the date of diagnosis form a breast sample to the date of death or last follow-up. Primary ENKTL-Breast was diagnosed based on the diagnostic criteria of primary breast lymphoma proposed by Wiseman and Liao, and later modified by Hugh et al. as follows: (1) adequate pathological material was available for review; (2) radiologically, the breast was considered as the primary site or site of major manifestation of the lymphoma; and (3) there was no prior documentation of a similar histological type of lymphoma other than that of the ipsilateral axillary nodes [29–31]. The cases, which did not fulfill the criteria of primary ENKTL-Breast, were defined as secondary ENKTL-Breast.

### Histological assessment

All tissue specimens were fixed with 10% formalin and embedded in paraffin after routine processing. Tissue Sects. (3–4 µm) were stained with hematoxylin and eosin for subsequent microscopic examination.

### Immunohistochemistry analysis

For the immunohistochemical analysis, the following lymphoma antibodies were used: cytoplasmic CD3 (cCD3, PS1, Dako, Glostrup, Denmark), CD20 (L26; Dako, Glostrup, Denmark), CD5 (4C7; Novocastra, Newcastle, UK), CD4 (RMA-0620; Maixin, Shenzhen, China), CD8 (C8; Maixin, Shenzhen, China), TIA-1 (2G9; Dako, Glostrup, Denmark), granzyme B (GZB01; Neomarkers, Fremont, CA, USA), and CD56 (123C3; Zymed, Guangzhou, China), CD30 (Ber-H2; Neomarkers, Fremont, CA, USA), and Ki-67 (M7259; Dako, Glostrup, Denmark). All immunostaining were performed as previously described [32], and appropriate positive and negative controls were employed. The positive rate of CD30 and Ki-67 (positive tumor cells/total tumor cells) was recorded.

### In situ* hybridization*

EBV status was evaluated using in situ hybridization with a digoxin-labeled oligonucleotide probe complementary to two EBER, namely, EBER-1 and EBER-2 (EBER1/2; Dako, NO. Y520001). This was performed as previously described [33].

### T-cell receptor gene rearrangement analysis

*TR* gene rearrangement was detected based on BIOMED-2 operating instructions. The analysis was performed as previously described [32].

## Supplementary Information

**Additional file 1: Table S1.** Clinical features of ENKTL-breast queried in the literature. **Table S2.** Composition of breast lymphoma cases between 2010 and 2020 in our institution.

## Data Availability

All data generated or analyzed during the current study are included in this published article.

## Abbreviations

ENKTL: Extranodal natural killer/T-cell lymphoma
EBV: Epstein–Barr virus
OS: Overall survival
EBER: EBV-encoded small RNAs
TR: T-cell receptor
HBV: Hepatitis B virus
