# Supplementary material for: Extranodal natural killer/T-cell lymphoma of the breast: a retrospective clinicopathological analysis of a consecutive 11-year case series
Source: Orphanet J Rare Dis. 2021 Nov 18;16:479. doi: 10.1186/s13023-021-02110-x (PMC8600691; doi:10.1186/s13023-021-02110-x)
Supplement: Supplementary file 1 — Additional file 1: Table S1. Clinical features of ENKTL-breast queried in the literature. Table S2. Composition of breast lymphoma cases between 2010 and 2020 in our institution. [file 13023_2021_2110_MOESM1_ESM.docx]

**Table s1. Clinical features of ENKTL-Breast queried in the literature**

| **Case#** | **Reference#** | **P/S** | **Age/Sex** | **sites involvement** | **Stage** | **Initial presented with breast mass** | **B-symptoms** | **Origin** | **Concomitant diseases** | **IHC and ISH** | **TR generearrangement** | **Follow-Up** |
| --- | --- | --- | --- | --- | --- | --- | --- | --- | --- | --- | --- | --- |
| 1 | [6] | S | 47/F | Breast, BM, skin, liver | IV | Yes | NA | NA | NA | IHC: NA  EBER+ | ND | NA |
| 2 | [6] | P* | 44/F | Breast | I | Yes | NA | NA | NA | IHC: NA  EBER+ | ND | NA |
| 3 | [7] | P* | 20/F | Right breast | ND | Yes | Yes | Asian | Heart transplantation | CD3+,CD5-,TIA-1+,CD56+;  EBER+ | P | Died  (1 mo) |
| 4 | [8] | P* | 41/F | Right breast. | I | No | Yes | Caucasian | Saline breast implant | CD3+,CD5-,TIA-1+,CD56+;  EBER+ | M | Alive  (8 moths) |
| 5 | [9] | P* | 40/F | Bilateral breasts and other multi-organ involvement† | IV | Yes | Yes | Caucasian | Hypothyroidism | CD3+,TIA-1+,CD56-;  EBER+ | P | Died  (5 mo) |
| 6 | [10] | S | 44/F | Left breast, lung | ND | Yes | NA | NA | None | CD3+, CD56+;  EBER- | P | Died  (18 mo) |
| 7 | [11] | S | 30/F | Left breast, nasal cavity | IV | Yes | No | Caucasian | None | CD3+,CD5-,TIA-1+,CD56+;  EBER+ | ND | NA |

†including kidney, stomach, duodenum, lungs, pleuralcavity, uterus and bone.

IHC, immunohistochemistry; ISH, in situ hybridization; EBER, EBV-encoded small RNAs; M, monoclonal; NA, not available. ND, not done. P*, primary; P, polyclonal.

**Table s2. Composition of breast lymphoma cases between 2010 and 2020 in our institution**

| Type of lymphoma | Number of cases | Proportion |
| --- | --- | --- |
| DLBCL | 176 | 77.2% |
| B-ALL/LBL | 11 | 4.8% |
| MALT lymphoma | 11 | 4.8% |
| ENKTL | 8 | 3.5% |
| ALK-ALCL | 4 | 22 (9.7%) |
| T-ALL/LBL | 3 |  |
| Burkitt lymphoma | 2 |  |
| FL | 2 |  |
| CLL/SLL | 2 |  |
| HIV-related lymphoma* | 2 |  |
| High grade B lymphoma | 2 |  |
| CHL-NS | 2 |  |
| PTCL-NOS | 1 |  |
| SPTCL | 1 |  |
| Myeloid sarcoma | 1 |  |
| Total | 228 | 100% |

ALK-ALCL, anaplastic large cell lymphoma, ALK―negative; B-ALL/LBL, B-lymphoblastic leukaemia/lymphoma; CHL-NS, nodular sclerosis classic Hodgkin lymphoma; CLL/SLL, Chronic lymphocytic leukaemia / small lymphocytic lymphoma; DLBCL, diffuse large B-cell lymphoma, NOS; ENKTL, extranodal NK/T-cell lymphoma; FL, follicular lymphoma; MALT lymphoma, extranodal marginal zone lymphoma of mucosa-associated lymphoid tissue; PTCL-NOS, peripheral T―cell lymphoma, NOS; SPTCL, subcutaneous panniculitis-like T-cell lymphoma.

* HIV-related lymphoma：one is HIV-related PTCL, the remain one is HIV-related plasmablastic lymphoma
